# Strain pattern of each ligamentous band of the superficial deltoid ligament: a cadaver study

**DOI:** 10.1186/s12891-020-03296-0

**Published:** 2020-05-09

**Authors:** Masato Takao, Satoru Ozeki, Xavier M. Oliva, Ryota Inokuchi, Takayuki Yamazaki, Yoshitaka Takeuchi, Maya Kubo, Danielle Lowe, Kentaro Matsui, Mai Katakura, Ankle Instability Group, Jorge Acevedo, Jorge Batista, Thomas Bauer, James Calder, Nuno Corte-Real, Christopher DiGiovanni, Eric Giza, Mark Glazebrook, Stéphane Guillo, Siu Wah Kong, Peter G. Mangone, Kentaro Matsui, Frederick Michels, Caio Nery, Xavier M. Oliva, Satoru Ozeki, Christopher Pearce, Anthony Perera, Hélder Pereira, Bas Pinenburg, Fernando Raduan, James W. Stone, Masato Takao, Yves Tourné, Jordi Vega, Jin Woo Lee, Hua Yinghui, Mark Glazebrook

**Affiliations:** 1 Clinical and research institute for foot and ankle surgery, 341-1, Mangoku, Kisarazu, Chiba, 292-0003 Japan; 2 Department of Orthopaedic Surgery, Dokkyo Medical University Saitama Medical Center, 2-1-50, Minamikoshigaya, Koshigaya, Saitama, Japan; 3 Department of Human Anatomy, University of Barcelona, Calle Casanova, 143, 08038 Barcelona, Spain; 4 Department of Health Services Research, Faculty of Medicine, University of Tsukuba, 1-1-1 Tenno-dai, Tsukuba, Ibaraki, Japan; 5 Tokyo National College of Technology, 1220-2, Kunugida-machi, Hachioji, Tokyo, Japan; 6 Department of Orthopaedic Surgery, Teikyo University, 2-11-1 Kaga, Itabashi, Tokyo, Japan; 7 Department of Orthopaedic Surgery, Lions Gate Hospital, North Vancouver, BC Canada; 8 Division of Orthopaedic Surgery, Dalhousie University, Queen Elizabeth II Health Sciences Center Halifax Infirmary (Suite 4867), 1796 Summer Street Halifax, Halifax, Nova Scotia B3H3A7 Canada

**Keywords:** Tibionavicular ligament, Tibiospring ligament, Tibiocalcaneal ligament, Superficial posterior tibiotalar ligament

## Abstract

**Background:**

There are few reports on the detailed biomechanics of the deltoid ligament, and no studies have measured the biomechanics of each ligamentous band because of the difficulty in inserting sensors into the narrow ligaments. This study aimed to measure the strain pattern of the deltoid ligament bands directly using a Miniaturization Ligament Performance Probe (MLPP) system.

**Methods:**

The MLPP was sutured into the ligamentous bands of the deltoid ligament in 6 fresh-frozen lower extremity cadaveric specimens. The strain was measured using a round metal disk (clock) fixed on the plantar aspect of the foot. The ankle was manually moved from 15° dorsiflexion to 30° plantar flexion, and a 1.2-N-m force was applied to the ankle and subtalar joint complex. Then the clock was rotated every 30° to measure the strain of each ligamentous band at each endpoint.

**Results:**

The tibionavicular ligament (TNL) began to tense at 10° plantar flexion, and the tension becomes stronger as the angle increased; the TNL worked most effectively in plantar flex-abduction. The tibiospring ligament (TSL) began to tense gradually at 15° plantar flexion, and the tension became stronger as the angle increased. The TSL worked most effectively in abduction. The tibiocalcaneal ligament (TCL) began to tense gradually at 0° dorsiflexion, and the tension became stronger as the angle increased. The TCL worked most effectively in pronation (dorsiflexion-abduction). The superficial posterior tibiotalar ligament (SPTTL) began to tense gradually at 0° dorsiflexion, and the tension became stronger as the angle increased, with the SPTTL working most effectively in dorsiflexion.

**Conclusion:**

Our results show the biomechanical function of the superficial deltoid ligament and may contribute to determining which ligament is damaged during assessment in the clinical setting.

## Background

The deltoid ligament has both superficial and deep layers consisting of up to six ligamentous bands [1]. The superficial layer of the deltoid ligament is composed of four ligamentous bands, including the tibionavicular (TNL), tibiospring (TSL), tibiocalcaneal (TCL), and superficial posterior tibiotalar (SPTTL) ligaments. Two ligamentous bands comprise the deep layer of the deltoid ligament: the deep anterior and posterior tibiotalar ligaments. Generally, the deltoid ligament is known to work cooperatively and is primarily responsible for 1) stabilizing the medial side of the ankle to limit anterior, posterior, and lateral translation of the talus and 2) restraining talar abduction at the talocrural joint [2, 3]. Specifically, the superficial deltoid resists eversion of the hindfoot, and the deep deltoid is the primary restraint to external rotation of the talus [4–7].

There are few reports in terms of detailed biomechanics of the deltoid ligament [8–12], and no studies have directly measured the biomechanics of each ligamentous band because of the difficulty in inserting sensors into the narrow ligaments.

Biomechanical data regarding each ligamentous band would contribute to precisely assessing which ligament is damaged in the clinical setting, leading to the design and performance of repair and reconstruction procedures before surgery. Thus, we used a Miniaturization Ligament Performance Probe (MLPP) system that can be inserted into small ligaments and allows for the precise measurement of the strain patterns of the deltoid ligament during ankle motion.

## Methods

### MLPP system

**Fig. 1:**
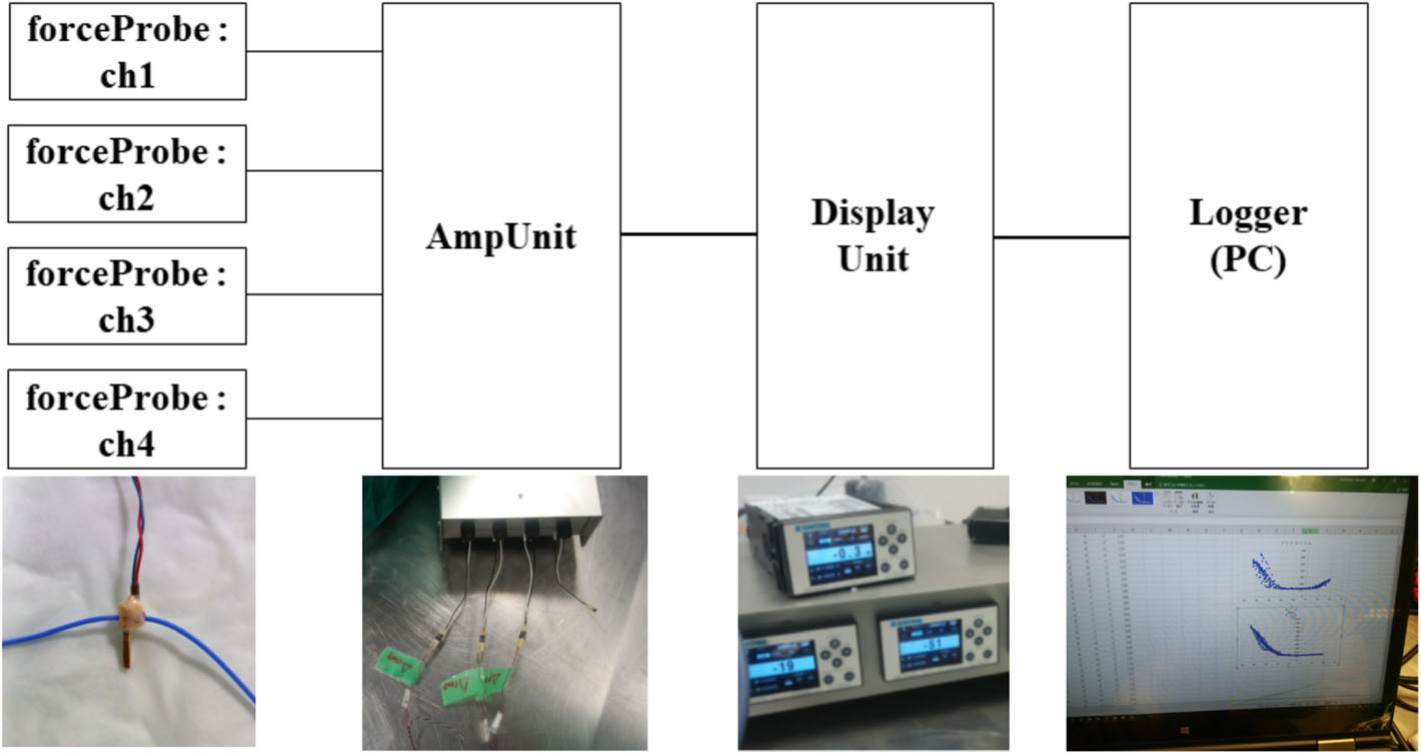
Miniaturization Ligament Performance Probe system. This system is composed of a force probe (left), an amplifier unit (middle left), a display unit (middle right), and a logger (right)

The MLPP system is composed of a strain gauge (force probe), an amplifier unit, a display unit, and a logger (Fig. 1). This system is capable of detecting small changes in resistance on the force probe. These changes in resistance are then enlarged by the bridge of an amplifier unit and transferred to the input of the display unit. After analogue-to-digital conversion by the display unit, the amount of strain is displayed. This strain measurement is converted to analogue, and its voltage is finally recorded in the logger.

**Fig. 2:**
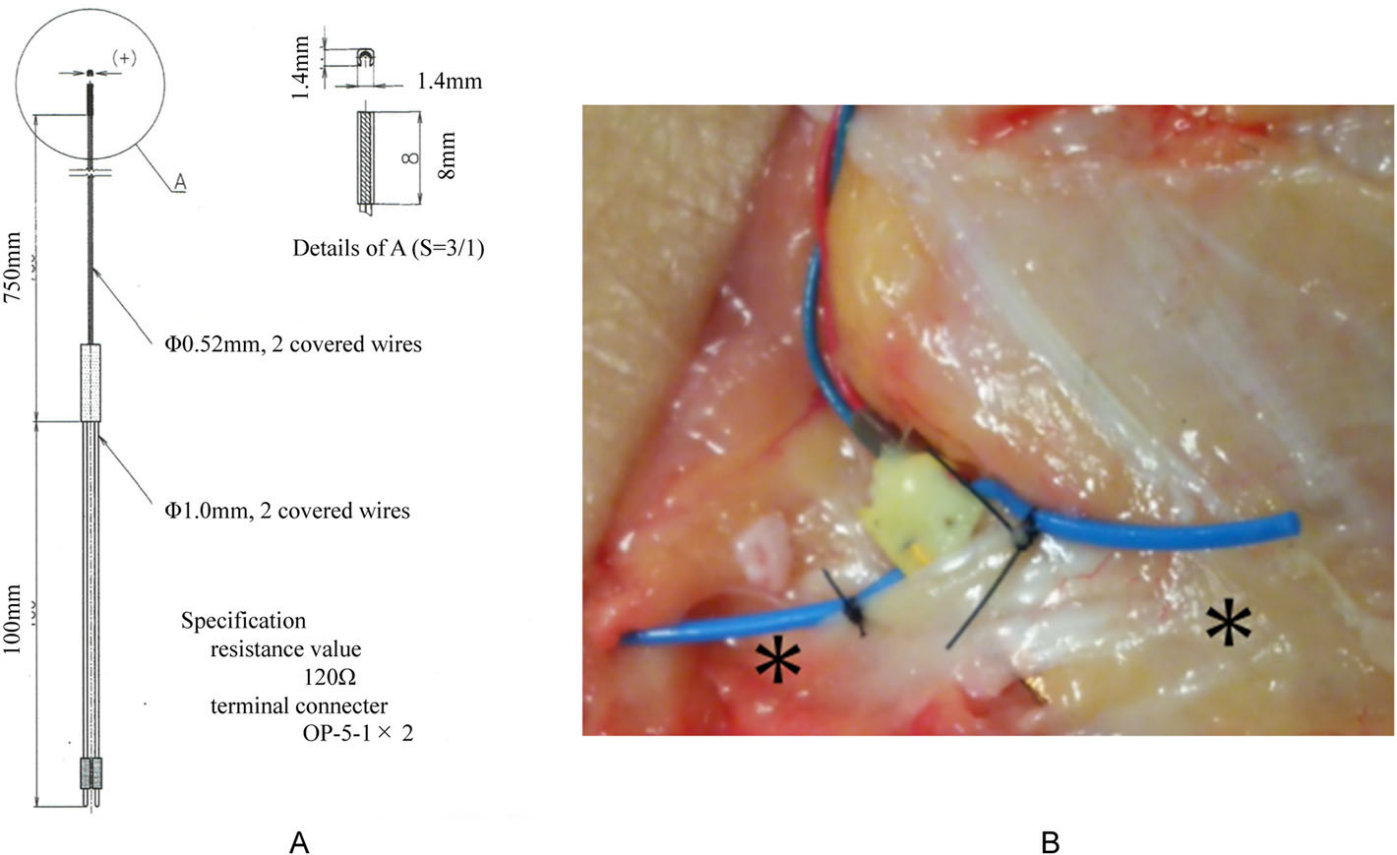
Force probe (strain gauge). The force probe is rectangular (**a**) and has a tubular structure with slits entering vertically on one side of its surface (**b**)

The force probe (Showa unilateral strain gauge; Showa Measuring Instruments Inc., Tokyo, Japan) is rectangular (width, 1.4 mm; height, 1.4 mm; length, 8 mm) and has a tubular structure with slits entering vertically on one side of its surface (Fig. 2a). When strain is applied to the force probe, the internal strain gauge is distorted, allowing the magnitude of strain to be measured. When the force probe is inserted into the tissue, it may rotate as forces are applied, causing the output to be reduced or inverted. To suppress this rotational influence, a tube was attached to the force probe, and both ends were sutured to the tissue to be measured (Fig. 2b).

**Fig. 3:**
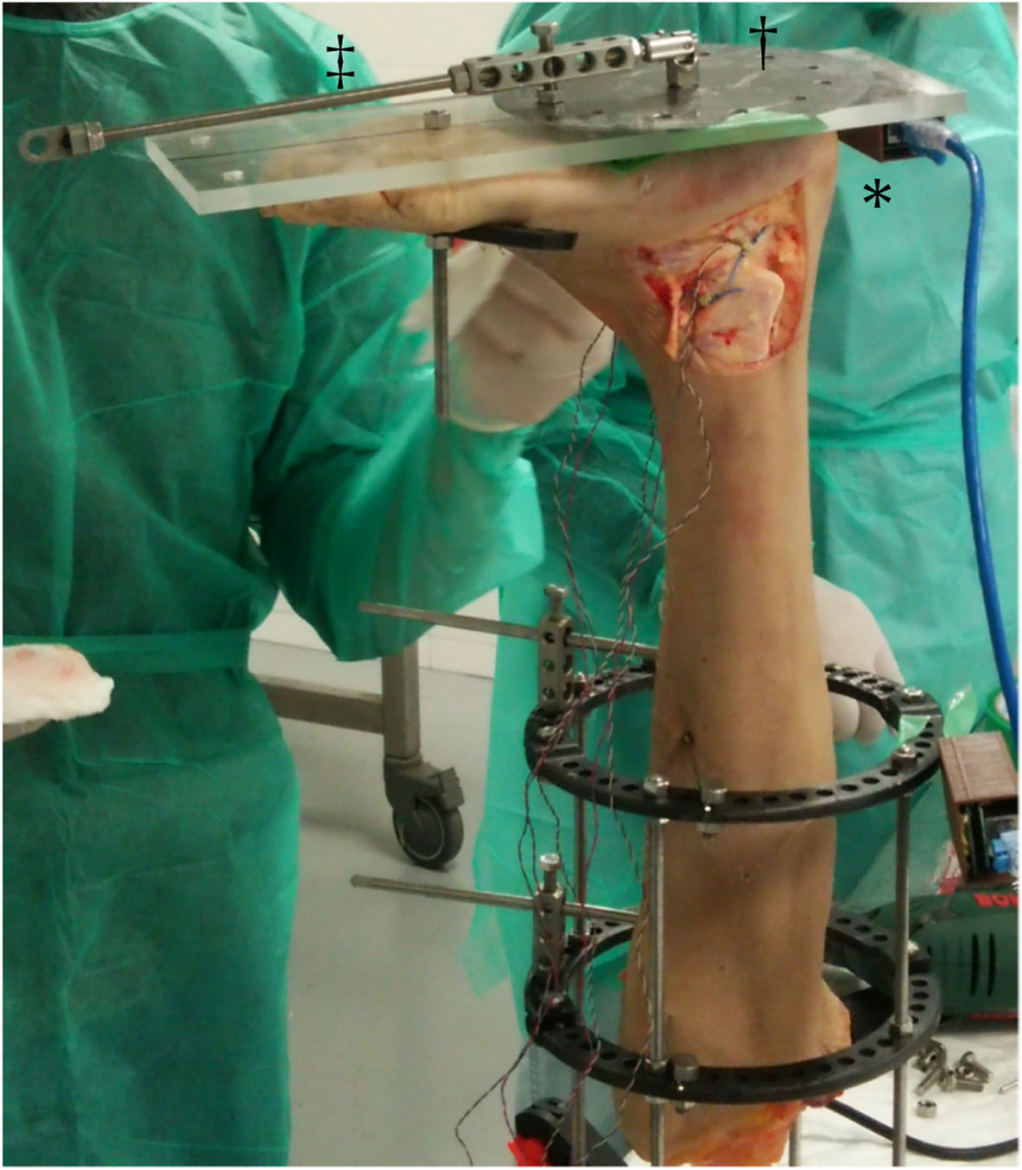
Setup of the specimen. The lower limb is fixed vertically to the measurement desk using an Ilizarov ring-shaped external fixator, and a performance cube (*), clock (†) and an arm (‡) are affixed to an acrylic plate

A performance cube was used to measure the position of the ankle (Fig. 3). The cube is composed of an MPU-9250 motion-processing sensor with a nine-axis sensor, an ESP32 microcontroller, and a logger. The MPU-9250 and ESP32 are loaded in the performance cube. The MPU-9250 is a sensor that acquires position information and can acquire values of motion in nine axes in total, each with angular acceleration, and geomagnetism. The MPU-9250 is equipped with hardware called a digital motion processor, which automatically performs measurements at the time of initialization of the sensor and calculates posture. The ESP32 is a microcontroller that calculates data obtained from the MPU-9250 and transmits data to the logger via a WiFi module. This performance cube is synchronized with the MLPP system.

### Cadaveric tests using the MLPP system

Six fresh-frozen through-the-knee lower extremity cadaveric specimens were used for this study (three right and three left). Three specimens were from men, and three were from women. The median age was 64 years (range 46–82 years). These specimens were free of ankle or hind foot deformities, did not undergo surgery or dissection, and did not have any history of trauma or other pathology that may alter the anatomy.

All cadaveric studies were performed at the University of Barcelona in Catalonia, Spain. All methods in this study were reviewed and approved by the Institutional Review Board of the University of Barcelona. Consent for the storage and use of the bodies for research purposes was given by all body donors before death or by their next of kin.

### Experiments on strain patterns of the superficial deltoid ligament

**Fig. 4:**
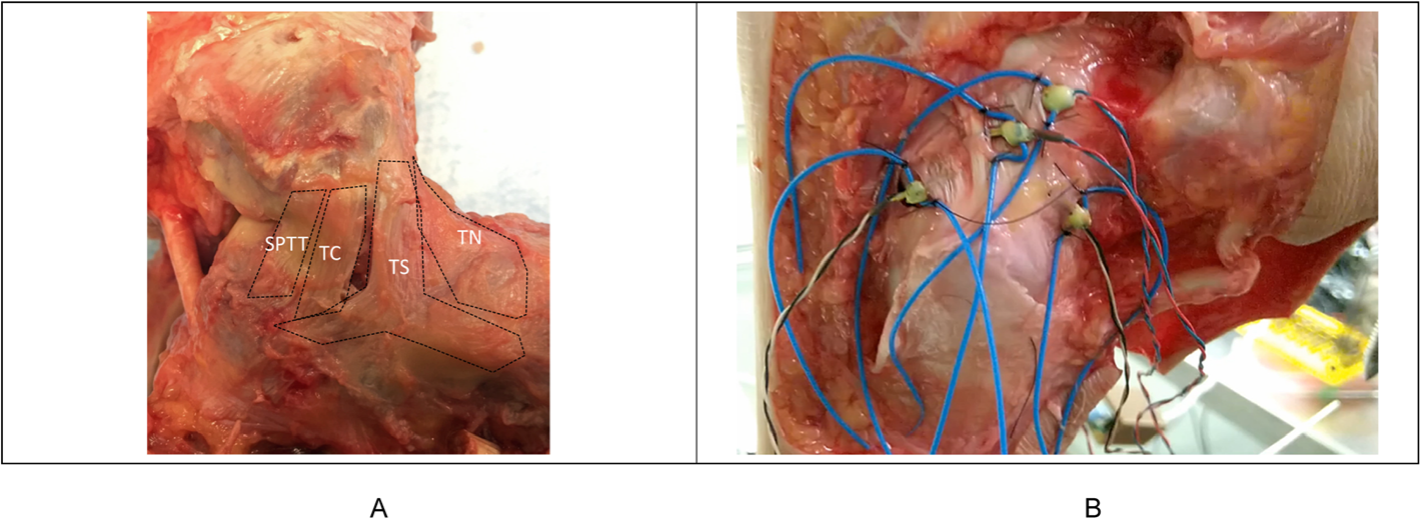
Tracing of each superficial deltoid ligament. Ligaments are not isolated in order to investigate them as one unit, and lines are drawn on the ligaments to trace each ligament from its origin to insertion on the bone (**a**). A force probe is placed in the mid-substance of each ligamentous band of the tibionavicular ligament, tibiospring ligament, tibiocalcaneal ligament, and superficial posterior tibiotalar ligament such that the slit of the force probe is aligned with the long axis of the ligament fibers (**b**)

The following procedures were performed in all specimens by a single experienced foot and ankle surgeon. An incision was made in the medial ankle, and the superficial layer of the deltoid ligament was exposed. Lines were drawn on the ligaments to trace each ligament from its origin to insertion on the bone (Fig. 4a). Ligaments were not isolated in order to investigate them as one unit. A force probe was placed in the mid-substance of each ligamentous band of the TNL, TSL, TCL, and SPTTL such that the slit of the force probe was aligned with the long axis of the ligament fibers (Fig. 4b). After introducing the force probe into the ligament, the force probe tube was sutured to the ligament fibers with 3–0 nylon thread to prevent the rotation of the force probe.

An Ilizarov ring-shaped external fixator was placed on the lower leg, and the lower limb was fixed vertically to the measurement desk using a vise to allow for the localization of the distal upper and proximal lower portions of the specimens. A round metal disk (clock, diameter 150 mm) with a 6-mm diameter hole every 30°, was affixed to an acrylic plate (width, 120 mm; length, 280 mm; thickness, 10 mm). The plate was fixed on the plantar aspect of the foot with a screw (diameter 6 mm) inserted into the calcaneus and a rod (diameter 8 mm) inserted between the second and third metatarsals (Fig. 3). This plate had a 25-cm arm where a 0.5-kg weight could be added at the end, applying a 1.2-N m force to the ankle and subtalar joint complex (0.5 kg × 0.25 m × 9.81 = 1.23 N m). This design resulted from a pilot study that used specimens to determine appropriate loading levels in order to achieve maximum range of motion where the plate would return to its original shape and length.

This arm was rotated every 30° on the clock to allow for measurement of the strain on each ligamentous band at various ankle positions. The ankle positions were defined as dorsiflexion with the arm at the 12 o’clock position, plantar flexion at the 6 o’clock position, inversion at the 3 o’clock position, and eversion at 9 o’clock position; in addition, 1 and 2 o’clock were defined as dorsiflexion-adduction, 4 and 5 o’clock were defined as supination (plantar flexion adduction), 7 and 8 o’clock were defined as plantarflexion- abduction, and 10 and 11 o’clock were defined as pronation (dorsiflexion-abduction).

After the investigation of strain in the designated ankle positions, the strain values of each ligament were also measured in axial motion of the ankle from maximal dorsiflexion to plantar flexion.

The angles of axial, sagittal, and horizontal motions were measured by an electronic goniometer (MPU-9250; TDK InvenSense, San Jose, CA, USA) synchronized to the MLPP system.

### Data analysis

The relationship between the foot positions and the tensile forces of each ligamentous band was analyzed. The tensile force data from the force probe were obtained by synchronizing the arm of the clock, which rotated every 30°, with the movement of the ankle from 15° dorsiflexion to 30° plantar flexion 10 times manually, and the strain of each ligamentous band during ankle motion was measured. Individual strain data were aligned to the value at neutral position (0) and to the maximum value (100). The average value at each position was connected by a line, and the ligament tension pattern was compared among the specimens.

## Results

### Tibionavicular ligament

**Fig. 5:**
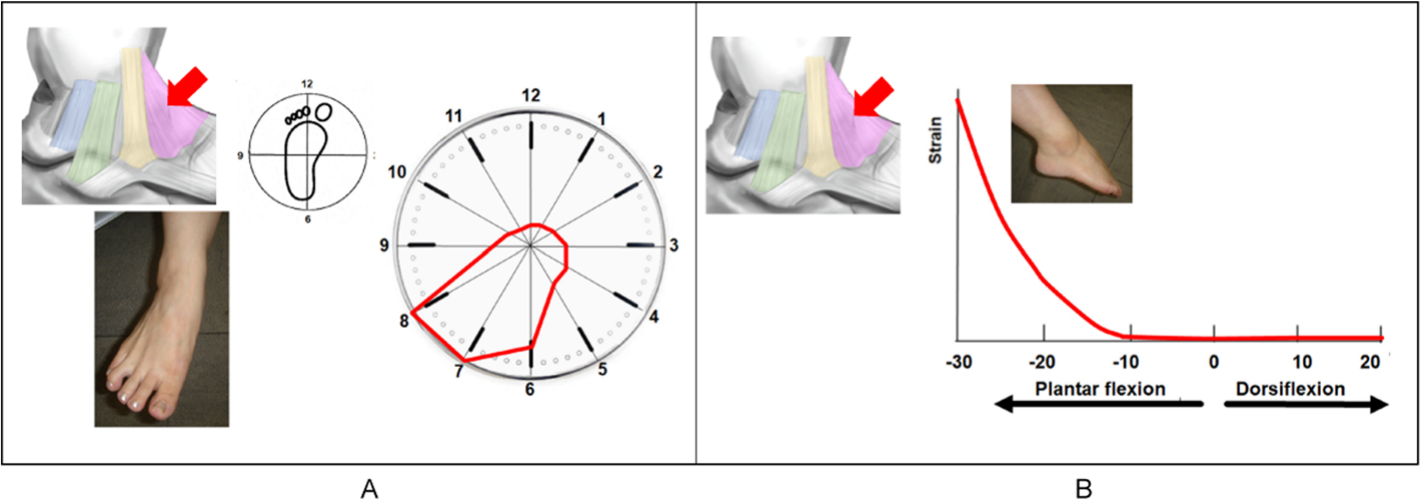
Strain pattern of the tibionavicular ligament (TNL). The TNL works most effectively in plantarflexion-abduction in clock motion (**a**). The TNL begins to tense gradually at 10° plantarflexion (**b**). The tension becomes stronger as the plantarflexion angle increases in axial motion

The TNL was under the most strain in plantarflexion-abduction (Fig. 5a). The TNL began to tense gradually at 10° plantarflexion. The strain became stronger as the plantarflexion angle increased to a maximum strain of 100 at 30° plantarflexion (Fig. 5b).

### Tibiospring ligament

**Fig. 6:**
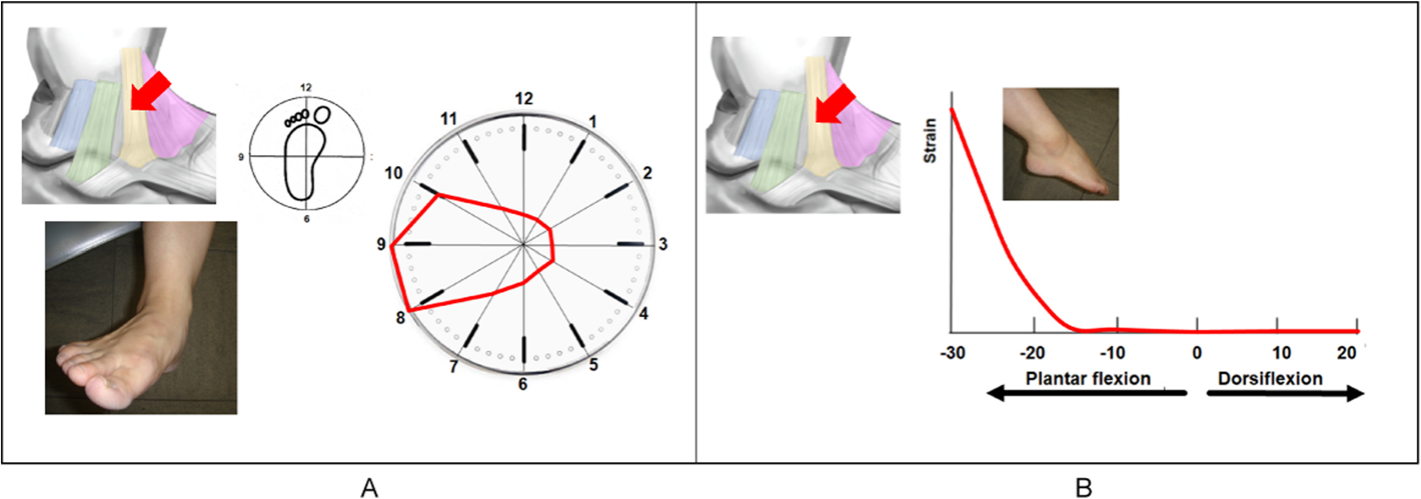
Strain pattern of the tibiospring ligament (TSL). The TSL works most effectively in eversion (**a**). The TSL begins to tense gradually at 15° plantarflexion, and the tension becomes stronger as the angle increases (**b**)

The TSL was under the most strain in eversion (Fig. 6a). The TSL began to tense gradually at 15° plantarflexion. The strain became stronger as the plantarflexion angle increased to a maximum strain of 100 at 30° plantarflexion (Fig. 6b).

### Tibiocalcaneal ligament

**Fig. 7:**
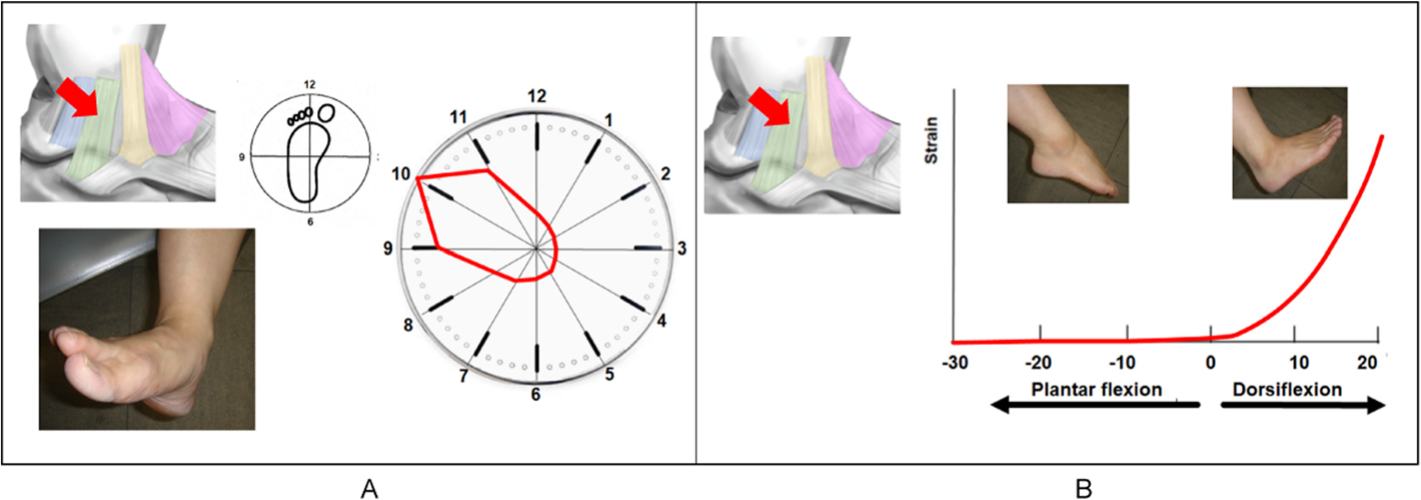
Strain pattern of the tibiocalcaneal ligament (TLC). The TCL works most effectively in pronation (**a**). The TCL begins to tense gradually at 0° dorsiflexion, and the tension becomes stronger as the angle increases (**b**)

The TCL was under the most strain in pronation (Fig. 7a). The TCL began to tense gradually at 0° dorsiflexion. The strain became stronger as the dorsiflexion angle increased to a maximum strain of 100 at 15° dorsiflexion (Fig. 7b).

### Superficial posterior tibiotalar ligament

**Fig. 8:**
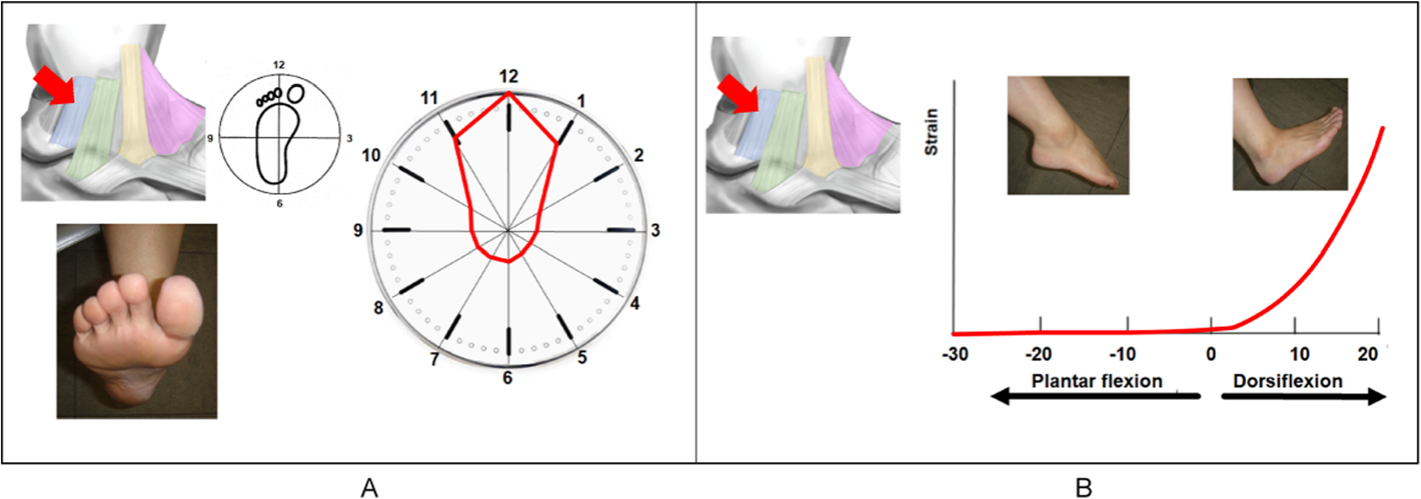
Strain pattern of the superficial posterior tibiotalar ligament (SPTTL). The SPTTL works most effectively in dorsiflexion (**a**). The SPTTL begins to tense gradually at 0° dorsiflexion (**b**). The tension becomes stronger as the angle increases

The SPTTL was under the most strain in dorsiflexion (Fig. 8a). The SPTTL began to tense gradually at 0° dorsiflexion. The strain became stronger as the dorsiflexion angle increased to a maximum strain of 100 at 15° dorsiflexion (Fig. 8b).

## Discussion

In this study, we gained a comprehensive understanding of the contribution of each ligamentous band in the deltoid ligament to overall ankle stability at various ankle positions.

Previous studies have evaluated biomechanics 1) by using a laboratory reference axis system to obtain a three-plane description of movements [13], 2) after sectioning each ligamentous band [5], 3) by using reluctance transducers to measure change in the deltoid ligament length [9], 4) by using computational models [10], and 5) by using a marker-based motion analysis [11]. This is the first study in which each ligamentous band of the deltoid was investigated without transection; thus, we precisely assessed how each ligament works.

We found that the TNL, TSL, TCL, and SPTTL work most effectively in plantar flexion-abduction, abduction, pronation (dorsiflexion-abduction), and dorsiflexion, respectively. In the clinical setting, by examining the range of pain, the physician could evaluate which ligament is damaged, and then the results would make it possible to determine whether surgery is necessary in accordance with the person’s life and exercise.

Repair of the deltoid ligament is now controversial. In addition, clear indications for operative repair have not yet been well established. Some studies have shown that repair of the ruptured ligament is beneficial and can produce satisfactory results [8, 12, 14, 15]. By contrast, other studies showed repair of the deltoid ligament to be unnecessary [6, 16–19]. There may be some advantages of adding deltoid ligament repair for patients with high fibular fractures or in patients with concomitant syndesmotic injury and fixation [20], but previous studies did not evaluate each ligamentous band by physical examination before surgery. In this study, we clarified the biomechanics of each ligamentous band of the deltoid ligament. This will allow for detailed preoperative assessment of ligament damage and adaptation of operative techniques and procedures, possibly leading to established indicators for operation.

### Limitations

The disadvantage of MLPP is that it measures the strain value of the ligament instead of tensile force. In the elastic range where the deltoid ligament can return to its original shape and length, force and strain showed a linear proportional relationship. Therefore, it is theoretically possible to convert the measured strain value to Newton force if Young’s modulus is obtained by calibration. However, it is difficult to accurately determine Young’s modulus because the water content of the tissue decreases with time and the elasticity of the ligaments changes. The acceptable variation in the results of this study might be influenced by temporal changes in the elasticity of the ligaments. In addition, the preservation process of fresh-frozen cadavers influenced elasticity.

## Conclusions

We demonstrated biomechanical properties of each ligamentous band of the superficial layer of the deltoid ligament. These findings provide a better understanding of the biomechanical function of the deltoid ligament, which could help in informing repair and reconstruction procedures.

## Data Availability

The datasets used and/or analyzed during the current study are available from the corresponding author on reasonable request.

## Abbreviations

MLPP: Miniaturization Ligament Performance Probe
SPTTL: Superficial posterior tibiotalar ligament
TCL: Tibiocalcaneal ligament
TNL: Tibionavicular ligament
TSL: Tibiospring ligament
